# Experimental Approaches to Diabetes Mellitus

**DOI:** 10.5152/eurasianjmed.2022.22304

**Published:** 2022-12-01

**Authors:** Muhammed Yayla, Damla Binnetoğlu

**Affiliations:** Department of Pharmacology, Kafkas University Faculty of Medicine, Kars, Turkey

**Keywords:** Diabetes, high-fat diet, mice, rat, streptozotocin

## Abstract

One of the most common health problems today, diabetes is a serious, chronic, and complex disease characterized by high blood glucose levels. Nowadays, experimental diabetes models are being developed to study existing diabetes in depth, to improve diabetes medications, or to develop new medications. The protocols developed to date to create an experimental diabetes model are finalized in different time intervals and depending on various factors. With these models, which can be designed in vivo and in vitro, a picture similar to type 1 and type 2 diabetes can be created. In this review, we aimed to present the methodology, advantages, and disadvantages of all currently used experimental diabetes models in the light of current literature.

**Main Points**

- İslet isolation and insulin secretion studies also shed light on islet transplantation studies.
- Insulin resistance models in vitro are growing in popularity.
- Type 2 diabetes-induced high-fat diet/streptozotocin model is the best way and has high similarity according to the clinical findings.

## Introduction

Diabetes mellitus (DM) is a serious, chronic, and complex disease characterized by high blood glucose levels due to ineffective use of the hormone insulin or insufficient production of the hormone insulin. Clinically, hyperglycemia results from the deficiency or insufficiency of insulin, the hormone that enables the conversion of circulating glucose into energy in the cell.^1^ Diabetes is divided into 4 subclasses: type 1, type 2, specific diabetes due to other causes (such as neonatal diabetes), and gestational diabetes.^2^ Today, the prevalence of DM is increasing. This situation, which is one of the most important health problems in the world, causes it to maintain its popularity. The World Health Organization reports that 6.4% of the adult population has diabetes. While 7.8% were projected to have DM in 2030, today this rate has exceeded expectations. Although the majority are diagnosed with type 2 DM, 8.3% of the population is thought to be individuals diagnosed with DM.^2,3^ Cardiovascular mortality and morbidity are increased by the presence of nephropathy, neuropathy, and retinopathy caused by the presence of DM. The presence of free oxygen radicals, changes in serum proteins, endothelial dysfunction, and changes in acute phase proteins produced from the liver play a role in the formation of these complications.^4^ To control DM and its complications, patient awareness in terms of components such as proper nutrition, regular exercise, blood glucose control, use of appropriate pharmacological treatment, and awareness of the effects and side effects of the treatment used is important for patient welfare. In particular, lifelong medication use is one of the most important components in terms of patient adherence to treatment.^5^ Expenditures on treatment and care for DM and its complications are increasing rapidly and seriously reducing the quality of life of individuals. Glycemic control of patients is essential to prevent long-term micro- and macrovascular complications.^6^ Currently, various pharmacologic agents have been developed to provide glycemic control. These agents lower blood glucose levels by inhibiting various glucose transporters and carbohydrate digestive enzymes and maintain lipid and glucose homeostasis through peroxisome proliferator-activated receptor activation. Glucose transporter (GLUT) and sodium–glucose co-transporter families stand out as the current approach because they are proteins involved in glucose transport.^7-9^ In this context, scientists continue to conduct research to further investigate the mechanisms of existing drugs and to develop new therapeutic approaches. The popularity of studies on DM and its complications also makes DM experimental methods popular. These experimental methods can be designed in vivo or in vitro and can be used specifically for DM subtypes. Each of the experimental DM models has both advantages and disadvantages. The experimental model occurs at different time intervals and depending on various factors.^10^ The aim is to bring together the DM experimental methods used today with an up-to-date perspective.

## In Vitro Diabetes Models

Experimental diabetes models mainly aim to prevent hyperglycemia, which forms the basis of diabetes. The digestion of carbohydrates from the food we eat and their passage into the bloodstream is the first step. Therefore, it is among the most common methods to evaluate the in vitro activities of enzymes responsible for the digestion of carbohydrates (such as alpha-glycosidase and alpha-amylase) in the first place of agents expected to have antidiabetic activity in studies. The conversion of physiological glucose to sorbitol and fructose leads to sorbitol accumulation in some tissues due to increased hyperglycemia in diabetic patients. Especially in tissues and cells such as lenses, nerves, kidneys, and erythrocytes, the lack of sorbitol dehydrogenase enzyme leads to sorbitol accumulation in these tissues and cells and leads to the development of diabetic complications. Therefore, agents that inhibit aldose reductase enzyme activity prevent sorbitol accumulation and are therefore an important parameter used in experimental studies. Glucagon-like peptide-1 (GLP-1) analogs, which are of great importance, have been used in the treatment of both diabetes and obesity in recent years. By inhibiting the dipeptidyl peptidase-4 (DPP-4) enzyme that degrades endogenous GLP-1, it increases GLP-1 levels, leading to glucose-dependent insulin secretion, delaying gastric emptying, and suppressing appetite. In studies evaluating antidiabetic activity, DPP-4 activity is now also tested. Demonstration of these enzyme activities in vitro are tests that should be guiding especially in plant-based studies before in vivo experimental studies, contributing to the study in both time and economic terms.

In vitro cell culture study is another important phase in which antidiabetic activity can be demonstrated before the use of experimental animals. In untreated patients with hyperglycemia, which forms the basis of diabetes, insulin resistance develops in peripheral organs and blood glucose cannot be used by target cells. Subsequently, increased insulin secretion leads to desensitization of beta cells after a while and the ability to secrete insulin is impaired. Some experimental models allow these steps to be performed in cell culture studies. In diabetic patients, insulin resistance is the most common problem in muscle, fat, and liver cells. Therefore, cells such as the C2C12 mouse myoblast cell, the 3T3-L1 mouse adipose cell, and the human liver cell are hyperglycemia-induced human hepatocarcinoma (HepG2) cells in which insulin resistance can be experimentally induced. They are the most preferred cells in the studies. Finally, experimental protocols are available to investigate the efficacy of antidiabetic agents on insulin secretion. Islet isolation from the pancreas can be performed in the first instance, and insulin secretion response can be tested in the appropriate environment. Insulinoma cells could instead be obtained and the effects of insulin secretion tested on them. The first part of this review brings together studies on the abovementioned systems.

## In Vitro Enzyme Activity

### Alpha-Glycosidase Activity

Glycosidases are a common group of enzymes that break the glycosidic bond between 2 saccharide molecules. Glycosidases play key roles in several important biological processes such as lysosomal catabolism of glycoconjugates, post-translational modification of glycoproteins, and intestinal digestion. Preparations of glycosidase inhibitors serve as effective tools to realize the biological property of glycoprotein and to investigate the reaction and structure mechanisms of glycosidases.^11^

Alpha-glucosidase released from intestinal cells hydrolyzes oligosaccharides and polysaccharides into monosaccharide units such as glucose and fructose in the small intestine. In humans, α-glycosidase inhibitors are used to control type 2 DM and hyperglycemia. These can reduce carbohydrate intake and prevent the development of postprandial hyperglycemia. The inhibitory effect of the enzyme is therefore considered as a model of experimental diabetes.12

The inhibitory effect of α-glucosidase was determined by partially modifying the previously described methods. All samples (20 μL), an enzyme solution (10 μL, 1 unit/mL), and potassium phosphate buffer (50 μL, 50 mM, pH 6.9) were mixed and incubated at 37°C for 5 minutes. After adding p-nitrophenyl-α-d-glucopyranoside (20 μL, 3 mM) as a substrate to start the reaction, the mixture should again be incubated at 37°C for 30 minutes. Following incubation, 0.1 M sodium carbonate (50 μL) should be added to stop the reaction. Acarbose is used as a positive control and results are obtained by measuring the amount of nitrophenol at 405 nm using a microplate reader. The percentage inhibition of all samples is calculated with the formula: Inhibition (%) = (1 − Δsample/Δcontrol) × 100.^13^

Another method used to evaluate the inhibition effect of α-glycosidase is to mix phosphate buffer (pH 7.4, 75 µL), 5 µL of different concentrations (10-30 µg/mL) of antidiabetic agents and α-glycosidase enzyme solution (20 µL) prepared in phosphate buffer (pH 7.4). After pre-incubation, 50 μL of p-nitrophenyl-d-glycopyranoside in phosphate buffer (pH 7.4, 5 mM) was added, the solution was re-incubated at physiological temperature (37°C), and the absorbance was measured at 405 nm.^14,15^

### Alpha-Amylase Activity

Recently, antioxidants are known to inhibit enzymes such as butyrylcholinesterase, acetylcholinesterase, α-amylase, carbonic anhydrase, and α-glycosidase, which are associated with diseases such as type 2 DM, Alzheimer's disease, and glaucoma. Alpha-amylase inhibitory property is therefore experimentally evaluated as a measure of antidiabetic efficacy.

Alpha-amylase inhibition effects 

In principle, 1 g starch should be dissolved in 50 mL NaOH solution (0.4 M) and heated at 80°C for 20 minutes. It should then be made up to 100 mL at pH 6.9 and using distilled water. Then, 35 μL starch solution, 35 μL phosphate buffer (pH 6.9), and 5 μL antidiabetic agent should be mixed. After incubation at 37°C for 20 minutes, 20 μL of enzyme solution should be added and incubated again for 20 minutes. The reaction should be completed by adding 50 μL of 0.1 M HCl and absorbance measured at 580 nm. One unit of α-amylase enzyme is the amount of enzyme that releases 1.0 mg of maltose from starch in 3 minutes at 20°C, pH 6.9.^16^

### Aldose Reductase Activity

Aldose reductase (AR), the first rate-limiting enzyme in the polyol pathway, belongs to the aldo-keto reductase superfamily and is a monomer containing 315 amino acid residues. This overproduction of AR and sorbitol dehydrogenase in the polyol pathway and depletion of reduced Nicotinamide adenine dinucleotide phosphate (NADP^+^) and oxidized Nicotinamide adenine dinucleotide (NAD^+^), cofactors of this process, cause various metabolic process disorders such as nephropathy, retinopathy, cataracts, and neuropathy. The aforementioned metabolic abnormalities are the primary targets of diabetic complications in tissues involved in insulin-independent glucose uptake and are responsible for early tissue damage in organs.

In order to uncover new AR inhibitors, the synthesis, characterization, and biological activity of a series of novel acyl hydrazones are being investigated.^17^

Aldose reductase activity is determined with 1 M Na-phosphate (pH 5.5), 0.11 mM reduced form of NADP^+^ (NADPH), and dl-glyceraldehyde (4.7 mM). The enzyme assay is based on a decrease in NADPH concentration at 340 nm.^18^

### Dipeptidyl Peptidase-4 Activity

The incretin hormones GLP-1 and glucose-dependent insulinotropic polypeptide (GIP) are released from enteroendocrine cells in the small intestine in response to the presence of nutrients. These hormones facilitate glucose regulation by stimulating insulin secretion in a glucose-dependent manner while suppressing glucagon secretion. In patients with type 2 DM, impaired insulin response to GLP-1 and GIP results in hyperglycemia. Dipeptidyl peptidase-4 inhibitors block the breakdown of GLP-1 and GIP to increase levels of the active hormone. Dipeptidyl peptidase-4 inhibitors have been shown to provide glycemic control in clinical trials.^19^

Dipeptidyl peptidase-4 inhibitor screening kit is used in the literature. Briefly, each of the samples (10 μL) with different concentrations in DMSO should be pipetted into a 96-well plate, and then diluted assay buffer (30 μL), a diluted human-recombinant DPP-4 enzyme solution (10 μL), and diluted fluorogenic substrate, Gly-Pro-aminomethylcoumarin (AMC) (50 μL) should be added. Sample should be added for negative and positive control. The 96-well plate should be incubated at 37°C for 30 minutes with shaking. After incubation, the fluorescence of the free AMC group resulting from the reaction should be monitored in an excitation wavelength range of 350-360 nm and an emission wavelength range of 450-465 nm using a microplate reader. The percentage inhibition is calculated using the following formula.

% inhibition = [(OD control − OD sample)/(OD control)] × 100%, where OD control is the absorbance of the negative control and OD sample is the absorbance of the sample.^20^

### Insulin Secretion

Nowadays, potassium channel blockers such as sulfonylureas are used in the treatment of diabetes-induced insulin secretion disorders in pancreatic beta cells. In normal physiology, potassium channels, which are the most basic step in insulin secretion, depolarize the membrane, and potassium channels close when glucose entering the cell with Glut-2 contributes to adenosine triphosphate (ATP) production. Calcium then enters the cell and insulin secretion is stimulated. Experimental studies may therefore include a sulfonylurea agent as a positive control for insulin secretion. Glucose-dependent insulin secretion is the most preferred method in insulin secretion experiments. But not all beta cells respond to glucose.

### Islet Isolation and Insulin Secretion In Vitro

Rat and mouse species are widely used in islet isolation studies.^21,22^ When the prices of the materials to be used in the experiment are evaluated, the most economical application is on mice. However, the small size of the mouse is the most important factor that makes the attempt difficult during the pancreas removal phase.

Studies do not recommend direct removal of the pancreas and enzyme exposure. This practice reduces the efficiency in the number of islets to be isolated. Therefore, collagenase enzyme solution prepared for better quality islet isolation is injected through the biliopancreatic duct. Besides, hemostatic clamps should be placed to the right and left of the opening of the duct into the duodenum. Then, 2 mL of cold enzyme solution is slowly administered through the biliopancreatic duct with the help of a 23G needle tip, and the pancreas is allowed to swell. The pancreas is carefully removed and placed in a 50 mL sterile tube containing serum-free medium. After 10-15 minutes in a 37°C water bath, serum medium is added and shaken vigorously by hand for 20 seconds. In this way, the pancreas is also mechanically broken down. Then, centrifugation is done.

The key part of separating islets is to create a density gradient. Solutions such as histopaque are used for this. Here, the solution should not be mixed, especially when adding histopaque before centrifugation. After centrifugation, the islet layer between the histopaque and the medium should be carefully transferred into a 50 mL tube. Histopaque removal is then performed. The islets are collected and counted with a pipette under the microscope.^23,24^

They were then kept in RPMI medium containing 10% fetal bovine serum (FBS) in a 5% CO_2_ incubator and the medium should be changed every other day. Staining with dithizone (zinc staining) can be performed to show the presence of insulin in the islets. For demonstrating the effects of the agents on insulin release, isolated islets are transferred to 24-well plates (at least 20 islets per well). Then, to test glucose-dependent insulin secretion, the groups were added substances in an Hank's Balanced Salt Solution (HBSS) solution containing 5.5 mM and 16.5 mM glucose. The medium should be collected 1 hour after administration of the substances and measured with the Insulin ELISA kit. The periods here vary (Table 1).^25-32^

### Insulin Secretion in Insulinoma Cells In Vitro

Islet isolation for in vitro insulin secretion is costly and difficult, which has led to the use of insulinoma cells for this purpose.^33-36^ Easy reproduction in culture and the ability to form secretion with glucose stimulation are its most important advantages.^37-39^

Nevertheless, since they are cancer cells, they are also the cells preferred in anticancer activity studies. Insulinoma cells of rat and mouse origin are currently preferred.^40^ Although human-derived cells such as EndoC-BH1 are now in use, they are not yet widespread.

The MIN6, INS-1, and INS1-E are the best insulin-secreting insulinoma cells, but these cells are very difficult to access even commercially. Therefore, cells such as Beta-tc-6 and RIN-5F, which are easier to culture and better to purchase, are becoming increasingly common. These cells also respond well to sulfonylureas. A review of the literature shows that there are many differences in the methodology of these cells used experimentally. Optimization studies should therefore be planned at the beginning of the experiment (Table 2).^34,41-45^

### Insulin Resistance

Insulin resistance is the receptor insensitivity that develops at the cell level against increased insulin secretion when hyperglycemia is not controlled. Insulin receptors are a family of tyrosine kinase receptors.^46,47^ Insulin receptor substrates 1 and 2 are the most important intracellular messengers mediating the action of insulin. Thanks to these substrates, PI3K/AKT/mTOR and RAS/MAPK activation occur and the physiological effects of insulin are realized.^48,49^ The activities of receptors and substrates and the activities of transport systems (such as Glut-1, -2, -3, and -4) that allow glucose to enter the cell are therefore among the most basic parameters.

### With Hyperglycemia-Induced Human Hepatocarcinoma Cell

In the diabetic population, the risk of developing liver cancer is 2 to 3 times higher and hyperglycemia is the major causal factor driving tumor cells to aggressive metabolic growth. Studies have been conducted by measuring HepG2 cell proliferation. Hyperglycemia-induced human hepatocarcinoma, a human hepatocellular carcinoma cell line, should be obtained as a cell line. This cell line is glucose-sensitive and should not be propagated at high concentrations. Growth of cells should be performed in a solution containing 10% FBS and an antibiotic or antimycotic solution containing streptomycin, amphotericin B, and penicillin, and 1 or 2 g/L glucose such as Dulbecco’s modified Eagle’s medium (DMEM) or EMEM. Agents such as high-dose insulin, high-dose glucose, high-dose insulin and glucose, palmitic acid, and tumor necrosis factor-alpha (TNF-α) are used to induce insulin resistance in HEPG2 cells. What matters here is the dose used and the duration of exposure.

The development of insulin resistance was tested by checking whether cells consume glucose in the presence of insulin. This is either based on glucose consumption in the cell medium or by fluorescence measurement of intracellular glucose using 2-NBDG, which is more accurate. Periodic acid–Schiff (PAS) staining can also be performed to show the change in glycogen stores in liver cells.^50^ There are many different methods in the literature (Table 3).^51-54^

#### With 3T3-L1 Cell

Adipose tissue secretes cytokines that mediate systemic effects on obesity and insulin resistance. The antidiabetic drugs used cause metabolic differences and differences in gene expression profiles in 3T3-L1 adipocyte cells. These cells are therefore used in experimental diabetes research. The important fact is that 3T3-L1 cells are fibroblast cells in the first place and that they are differentiated into adipose tissue.

The mouse pre-adipocyte 3T3-L1 cell line is cultured in DMEM supplemented with 10% bovine calf serum, 100 units/mL penicillin, and 100 μg/mL streptomycin and used for research. Cells should be incubated at 37°C in 5% CO_2_. They were kept for 48 hours for proliferation and differentiation in a medium supplemented with 10% FBS (Invitrogen), 1 μM insulin, 0.5 mM 3-isobutyl-1-methylxanthine (IBMX), and 0.5 μg/mL dexamethasone. This should be followed by 2 more days in media containing 1 μM insulin.^55^ Excess palmitic acid is favored to induce insulin resistance, which is also related to the presence of adipose tissue in 3T3-L1 cells. Still, agents such as insulin, TNF-α, and dexamethasone can be used to induce insulin resistance in studies (Table 4).^56-59^

The development of insulin resistance was tested by checking whether cells consume glucose in the presence of insulin. This is either based on glucose consumption in the cell medium or by fluorescence measurement of intracellular glucose using 2-NBDG, which is more accurate. Oil red O staining for lipid accumulation in fat cells is a gold standard test.

#### With C2C12 Cell

Antioxidant substances were found to ameliorate palmitate-induced insulin resistance in C2C12 myocytes and 3T3-L1 adipocytes. This cell line is therefore used in research. The C2C12 mouse skeletal muscle cell lines must be obtained to establish cell culture and must be differentiated (from myoblast to myotubule cells) to be used experimentally. Dulbecco’s modified Eagle’s medium, 10% FBS, 100 units/mL penicillin, and 100 μg/mL streptomycin should be supplemented. Cells should be supplemented with 2% horse serum to initiate differentiation. All cells should be incubated at 37°C with 5% CO_2_.^60^ Myokines suppress lipid-induced inflammation, leading to attenuation of insulin resistance in mouse skeletal muscle. Therefore, appropriate regulation of inflammation may improve lipid-mediated insulin resistance in skeletal muscle cells (Table 5).^61,62^ The development of insulin resistance was tested by checking whether cells consume glucose in the presence of insulin. This is either based on glucose consumption in the cell medium or by fluorescence measurement of intracellular glucose using 2-NBDG, which is more accurate.

### In Vivo Diabetes Models

Both type 1 and type 2 DM are multifactorial diseases in which a very complex genetic architecture interacts with environmental factors to contribute to disease development. Early studies were based on modeling diabetes in dogs by removing part or the entire pancreas. Today, DM can be modeled in many animal species by chemical, surgical (pancreatectomy), viruses, and genetic alterations. Mice and rats are more commonly used in diabetes models, as in other experimental models.

### Models of Type 1 Diabetes

Type 1 diabetes can be modeled by a variety of different mechanisms to reduce insulin production, ranging from chemical ablation of beta cells to spontaneous generation of autoimmune diabetic rodents. The most commonly used and accepted model is the chemical-induced model. The most preferred model of type 1 diabetes is the streptozotocin-induced model. Rats and mice are more frequently preferred as experimental animals.[Table t6-eajm-54-S1-s145]

### Streptozotocin-Induced

treptozotocin (STZ), a metabolite of *Streptomyces achromogenes*, was discovered in 1959. A study conducted discovered that STZ causes diabetes after intravenous administration. Streptozotocin is suggested to exert its diabetes-inducing effect, which is also proven by histological examinations, only by inhibiting insulin secretion without necrosis of β cells. In a study monitoring β cells after STZ administration by electron microscopy, it was determined that STZ did not necrosis β cells but exerted its effect by degranulating β cells. Thanks to the glucose molecule in its structure, STZ inhibits insulin secretion by binding to glucoreceptors in the plasma membrane.^63^

Alloxan and STZ are considered the most potent diabetogenic chemicals ever used in diabetes research. Both chemicals are used as cytotoxic glucose analogs that tend to accumulate in pancreatic β cells that secrete insulin via the glucose transporter (GLUT2). Streptozotocin is preferred to alloxan because it is less toxic and more stable. Streptozotocin acts as a nitrozourea analog. The mechanism of action of the toxicity of STZ is related to the DNA-alkylating effect of its methylnitrozourea component. Methyl group transfer from STZ to the DNA molecule causes damage and leads to DNA fragmentation.^64^

The first 3 days are the most important stage in the STZ-induced diabetes model. Due to beta-cell destruction, insulin released leads to severe hypoglycemia and the death of the experimental animal. Dextrose should be added to their water for the first 3 days to prevent this. It should be followed up in the first 24 hours and isotonic should be given orally and intraperitoneally if necessary. At the end of the third day, animals with fasting blood glucose above 200 mg/dL can be considered diabetic.

Streptozotocin-induced diabetes is more commonly recognized as type 1. Blood glucose and weight should therefore be monitored regularly in these studies. It is important to keep the number of experimental animals in the number of groups high. Experiment duration can last from 15 days to 6 months depending on the model to be built. There are various protocols to induce diabetes in mice using STZ. Mice, especially Balb/c, are more resistant to STZ than rats. On average, up to 200 mg/kg is given. In the first protocol, STZ is administered in low doses but repeatedly. This results in less damage to the islets of Langerhans but more loss of activity of β cells. This method, commonly used for the induction of type 1 diabetes, is based on intraperitoneal administration of STZ at a dose of 40 mg/kg for 5 days. Another model of diabetes-induced using STZ involves a single administration of high-dose STZ. The high dose of STZ produces a pattern of diabetes within 48 hours, but it should be noted that this dose is highly toxic to β cells in the pancreas. Although this protocol, which can raise blood glucose levels above 500 mg/dL, is more toxic than multiple STZ doses, it is still highly preferred because it achieves the desired result in a single injection. It was further reported that STZ 65 mg/kg intraperitoneally was administered to evaluate diabetic agents and to investigate the pathogenesis of type 1 diabetes. In another protocol, nicotinamide is given simultaneously with STZ administration. Nicotinamide partially protects against STZ toxicity. In addition to approximately 60% loss of β-cell function, this protocol produces more stable and moderate hyperglycemia.^63,65^

To create an STZ-induced DM model, rats weighing 140 to 300 g were administered a single dose of STZ (35-70 mg/kg) dissolved in 0.1 M citrate buffer (pH 4.5) by intraperitoneal injection after an overnight fast. Control rats of the same age are injected with citrate buffer only. Streptozotocin is used as a model of type 1 diabetes because it causes damage to all β cells^,66-74^ (Table 6) 75 ^-^ 82

### Models of Type 2 Diabetes

To understand the pathophysiology and complications of type 2 diabetes, numerous animal models have been developed.^83-86^ These animal models include models of insulin resistance and/or models of beta cell failure. On the other hand, many animal models of type 2 diabetes use obese animals. It better reflects the situation in humans as obesity is closely associated with the development of type 2 diabetes. In most cases, these models are genetic models with obesity, glucose intolerance, and/or insulin resistance leading to high blood glucose levels. Both the development and progression of diabetic complications are influenced by several factors, including obesity, insulin resistance, hyperglycemia, and hyperlipidemia.^87^ The most preferred model of type 2 diabetes is the high-fat diet (HFD) and low-dose STZ-induced model. It can be induced in both rats and mice. The most important factor in the choice of experimental animal species is cost. At the same time, the choice of an HFD is of great importance in these models. Studies on fatty diets generally favor diets containing 45%-60% fat. A 60% fat diet model can be chosen if the model needs to be developed in a shorter period, if the animals need to be fed, cared for, etc., and if cost is a constraint.^88^

### High-Fat Diet and Streptozotocin-Induced

Low-dose STZ (30-40 mg/kg), which causes partial damage to β cells, can sometimes be administered in combination with repeated doses and an HFD (containing an average of 40%-60% fat) to create a model of type 2 diabetes.^89^ Animals that develop obesity after being fed an HFD for a certain period are given low-dose STZ to cause partial destruction of pancreatic beta cells, thus creating a model that may be close to the physiopathology of human type 2 diabetes.^83-85,87^

When the literature is examined, C57BL/6J was preferred as a mouse strain, especially in the HFD/STZ-induced type 2 diabetes model. It is therefore very important to monitor blood glucose, HbA1c, and weight during the experiment. Again, it is important for the compatibility of the model that the increase in cholesterol levels associated with obesity is shown in total cholesterol, low density lipoprotein (LDL), triglyseride (TG), and high density lipoprtoein (HDL) levels. Depending on the organ to be studied, the demonstration of glucose transports such as insulin receptors, insulin receptor substrates, Glut-1, -2, -3, and -4 by molecular or biochemical techniques constitute the most basic stages of the study.^90,91^ In these studies, obesity develops due to an HFD, unlike the type 1 diabetes model (Table 7).^92-100^

## Conclusion

Diabetes is a disease that remains unpopular because all its mechanisms are still unclear and there are many questions about it. Different methods have been developed and used up to the present to experimentally induce diabetes. The advantages and disadvantages of each method should be selected by considering the specific area to be elucidated, and the strategy of the study should be determined accordingly. Each method’s advantages and disadvantages should be selected by considering the specific area to be elucidated and the strategy of the study should be determined accordingly. In conclusion, it is clear from this review that experimental diabetes can be induced by various methods in studies designed to advance medicine.

## Figures and Tables

**Table 1.** Islet Isolation Procedure Differences

Animal Type	Enzyme	Medium	Time	Reference
Balb/c mice	Collagenase p	RPMI-1640		25
C57BL/6	Collagenase 4	RPMI-1640	10-15 minutes	26
Swiss albino mouse	Collagenase XI	RPMI-1640	15 minutes	27
BALB/c	Collagenase I and II	CMRL-1066	14 minutes	28
C57BL/6	Collagenase V	RPMI-1640	15 minutes	29
SD rat	Collagenase 4	RPMI-1640	10-15 minutes	26
W rat	Collagenase XI	RPMI-1640		30
LEW rat	Collagenase V	RPMI-1640	20-25 minutes	31
Rat	Collagenase IV	RPMI-1640	4 minutes	32

**Table 2.** Insulin Secreted Insulinoma Cell Lines

Cell Line	Source	Glucose Transporter	GSIS	Reference
MIN6	Mouse insulinoma	Glut-2	Yes	34
INS-1	Rat insulinoma	Glut-2	Yes	41
INS1-E	Rat insulinoma	Glut-2	Yes	42
Beta-TC 6	Mouse insulinoma	Glut-2	Yes	43, 44
RIN-5F	Rat insulinoma	Glut-2	Yes	45

**Table 3.** Insulin Resistance Procedures in HepG2 Cell Lines

Substance Used	Dose	Duration	Additional Information	Reference
Glucose + insulin	25 mmol + 10^−5^ M	24 hours	Low glucose DMEM	51
Palmitic acid	0.25 mM	24 hours	DMEM	52
Glucose	30 mM	48 hours	Low glucose EMEM	53
Insulin	10^−6^ M	48 hours	DMEM	54

DMEM, Dulbecco’s modified Eagle’s medium.

**Table 4.** Insulin Resistance Procedures in 3T3-L1 Cell Lines

Substance Used	Dose	Duration	Additional Information	Reference
TNF-α	10 ng/mL	5 days	DMEM	56
Palmitic acid	0.5 mM	24 hours	DMEM	57
Dexamethasone	1 μM	72 hours	DMEM	58
Insulin	150 nM	5 days	DMEM/F12	59

DMEM, Dulbecco’s modified Eagle’s medium; TNF-α, tumor necrosis factor-alpha.

**Table 5.** Insulin Resistance Procedures in C2C12 Cell Lines

Substance Used	Dose	Duration	Additional Information	Reference
Palmitic acid	200 μM	24 hours	DMEM + horse serum	61
Palmitic acid	500 μM	8 hours	DMEM + horse serum	62

DMEM, Dulbecco’s modified Eagle’s medium.

**Table 7.** HFD/STZ-Induced Type 2 Diabetes Model in Rats and Mice

Type	Diet	Duration of Diet Before STZ	STZ Dose	Reference
SD rat	58% Fat	3 months	30-35 mg/kg	92
SD Rat	59% Fat	8 weeks	25 mg/kg	93
SD Rat	40% Fat	2 weeks	35 mg/kg	94
W rat	40% Fat	2 weeks	40 mg/kg	95
W rat	22% Fat	4 weeks	30 mg/kg	96
C57BL/6 mice	58% Fat	12 weeks	40 mg/kg × 5 days	97
C57BL/6 mice	60% Fat	2 weeks	60 mg/kg	98
ICR mice	60% Fat	8 weeks	50 mg/kg	99
C57BL/6 mice	45%	4 weeks	40 mg/kg × 2 days	100

HFD, high-fat diet; SD, Sprague–Dawley; STZ, streptozotocin; W, Wistar.

**Table 6. t6-eajm-54-S1-s145:** STZ-Induced Type 1 Diabetes Model in Rats and Mice

Type	STZ Dose	Experiment Duration	Reference
C57BL/6J mice	50 mg/kg/day for 5 days	16 hours	75
Kunming mice	50 mg/kg/day for 5 days	10 hours	76
Balb/c mice	100 mg/kg	14 days	77
Balb/c mice	200 mg/kg	14 days	78
SD rat	35 mg/kg	6 weeks	79
Rat	90 mg/kg	3 months	80
SD rat	50 mg/kg	3 months	81
SD rat	60 mg/kg	30 days	82

SD, Sprague–Dawley; STZ, streptozotocin.
